# Coffee and Arterial Hypertension

**DOI:** 10.1007/s11906-021-01156-3

**Published:** 2021-08-09

**Authors:** Stanisław Surma, Suzanne Oparil

**Affiliations:** 1 Faculty of Medical Sciences in Katowice, Medical Univeristy of Silesia in Katowice, Medyków 18, 40-752 Katowice, Poland; 2 Club of Young Hypertensiologists, Polish Society of Hypertension, Warsaw, Poland; 3 Department of Medicine, School of Medicine, University of Alabama at Brimingham, Brimingham, AL USA

**Keywords:** Coffee, Caffeine, Blood pressure, Arterial hypertension

## Abstract

**Purpose of Review:**

Coffee is a very popular drink and an estimated 2.25 billion cups worldwide are consumed daily. Such popularity of coffee makes it the most consumed drink next to water. Numerous studies have shown a beneficial effect of habitual and moderate coffee consumption on the functioning of the nervous, digestive, and cardiovascular systems, as well as on kidney function. Taking into account the very high prevalence of arterial hypertension in the world (31.1% of adults), much controversy has been raised about the influence of coffee consumption on blood pressure and the risk of arterial hypertension. Moreover, there have been extensive discussions about the safety of coffee consumption for hypertensive persons.

**Recent Findings:**

There are over 1000 chemical compounds in coffee. The best characterized of these are caffeine, chlorogenic acid, trigonelline, kahweol, cafestol, ferulic acid, and melanoidins. These compounds have bidirectional influences on blood pressure regulation. The results of numerous studies and meta-analyses indicate that moderate and habitual coffee consumption does not increase and may even reduce the risk of developing arterial hypertension. Conversely, occasional coffee consumption has hypertensinogenic effects. Moderate habitual coffee consumption in hypertensive persons does not appear to increase the risk of uncontrolled blood pressure and may even reduce the risk of death from any cause.

**Summary:**

Moderate and habitual consumption of coffee (1-–3 cups / day) does not adversely affect blood pressure in most people, including those with arterial hypertension.

## Introduction

Coffee is the most consumed drink for humans next to water. According to the National Coffee Association USA, about 2.25 billion cups are drunk worldwide every day, totaling about 500 billion cups/year [1]. The available data indicate that the inhabitants of Finland consume the most coffee—on average over 10 kg per capita/year [2]. In the USA and Poland, coffee consumption is 4.4 kg and over 3 kg per capita/year, respectively [2]. Results of numerous studies indicate beneficial effects of regular moderate coffee consumption on the nervous, cardiovascular (CV), and digestive systems, as well as on kidney function [3–7]. Recent research findings indicate that regular consumption of 2–3 cups of coffee a day reduces the risk of nonfatal and fatal CV diseases, type 2 diabetes, endometrial cancer, and melanoma and non-melanoma skin cancer in the US population [8]. There are reviews of the literature that summarize the knowledge about the impact of coffee consumption on global health [9]. In contrast, the effects of coffee consumption on blood pressure (BP) and the risk of arterial hypertension are controversial. Given the very high prevalence of arterial hypertension (31.1% of adults worldwide) [10] and the significant influence of diet on its pathogenesis, this article reviews the impact of coffee consumption on the risk of its occurrence.

## Biologically Active Compounds in Coffee and Their Effects on BP

It is estimated that there are over 1000 chemical compounds in coffee [9]. The composition of coffee depends on many factors, including the type of coffee (e.g., *Coffea arabica*, *Coffea canephora*, *Coffea liberica*) (Table 1), the method of production (wet, dry, semi-dry/semi-wet, and bio-processing), and the method of preparation (e.g., traditional coffee, espresso). Pre-harvest factors (e.g., sunlight) and post-harvest factors (e.g., method of processing coffee beans) account for approximately 40% and 60% of the organoleptic (being perceivable by the senses, such as smell, appearance, taste, and touch), physical, and biochemical properties of coffee, respectively [5, 9, 11–13].

**Table 1:**
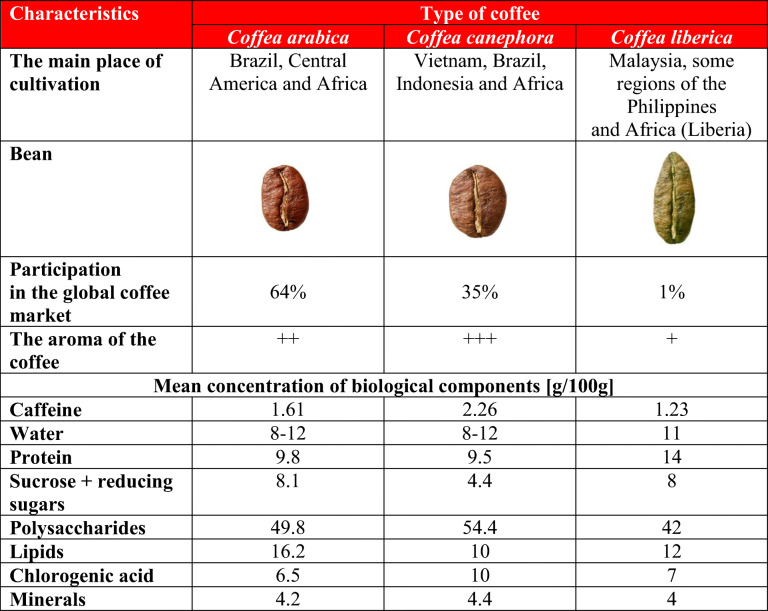
Characteristics of *Coffea arabica*, *Coffea robusta*, and *Coffea liberica* [14–16]

**Figure 1:**
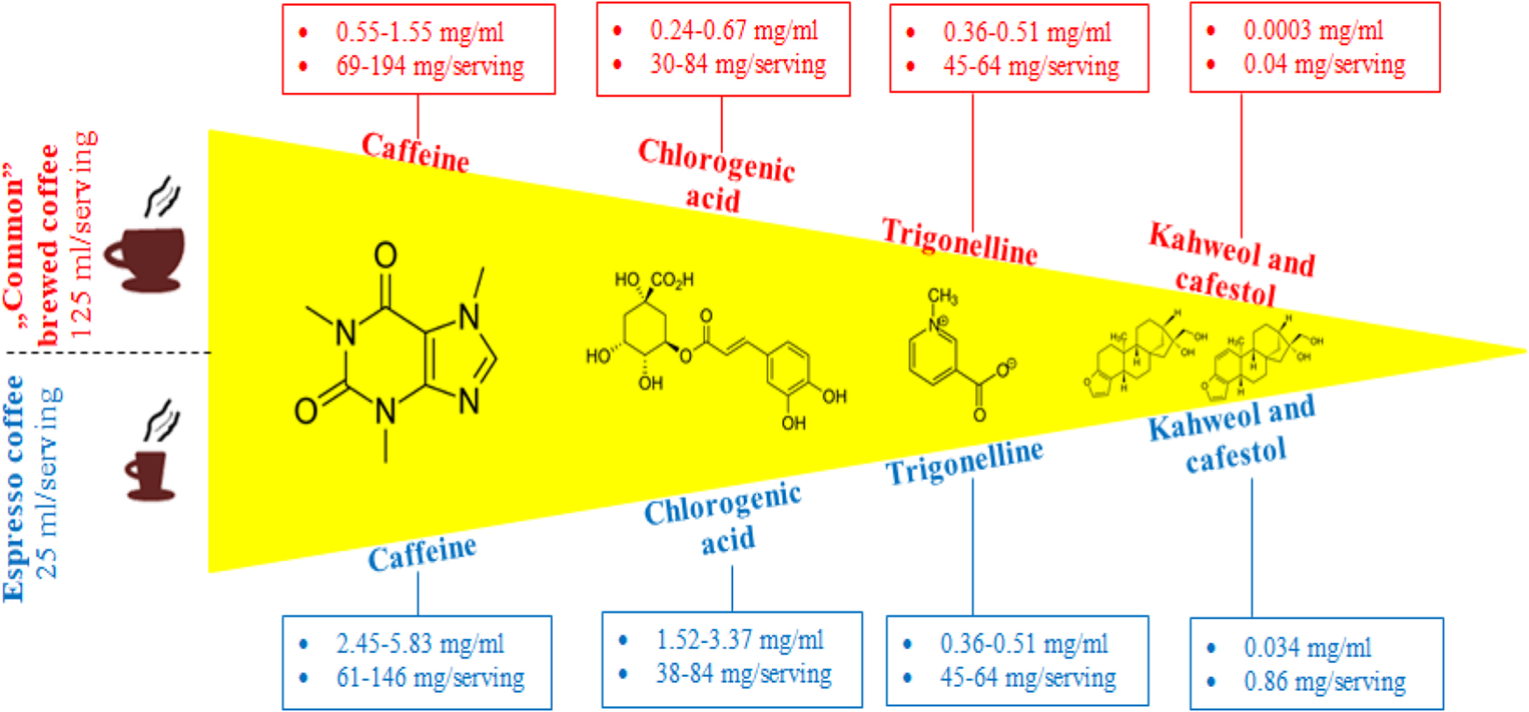
The main chemical compounds found in traditional coffee and espresso in terms of concentration and total amount in the usual serving sizes [3]

**Figure 2:**
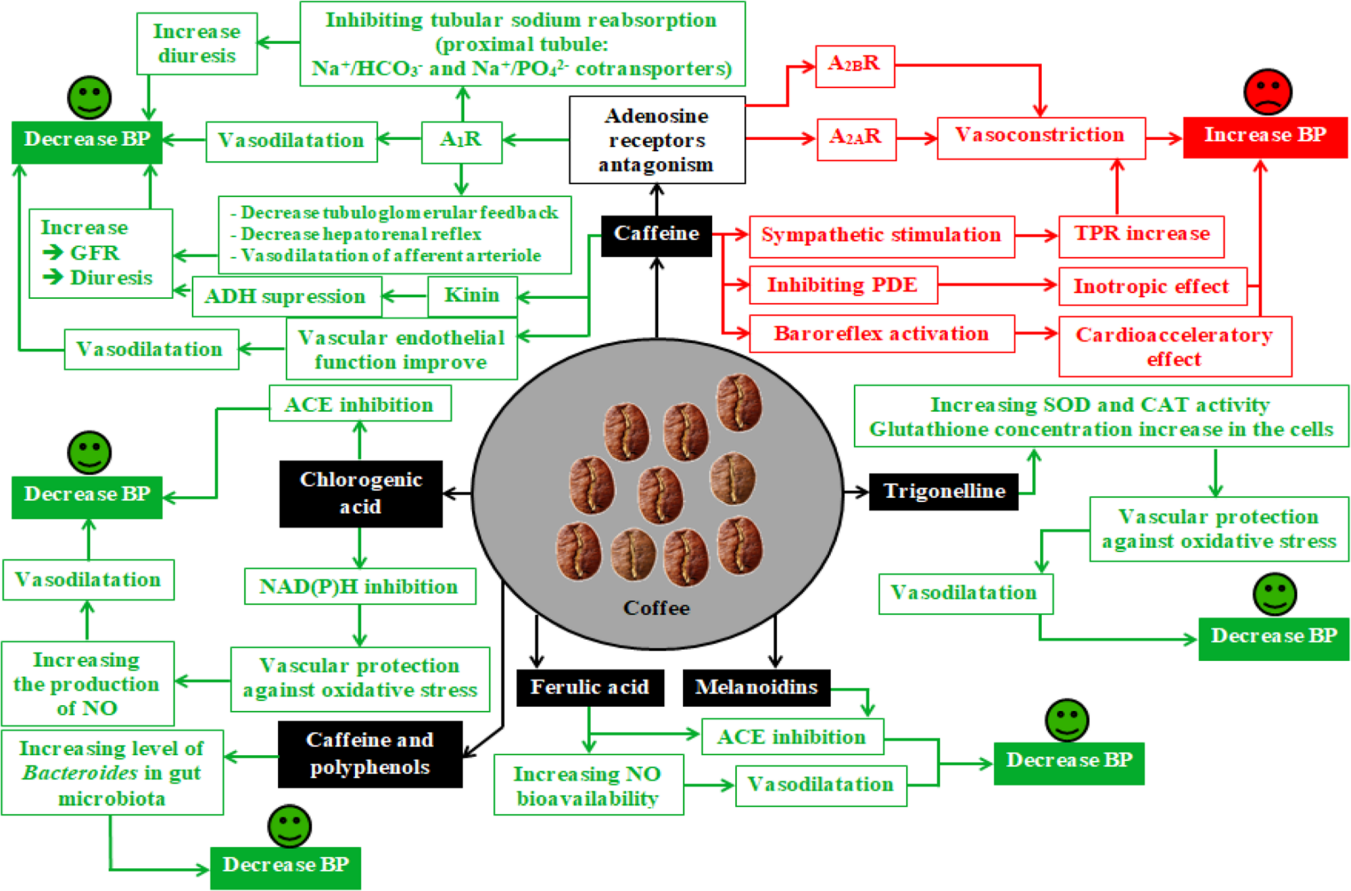
Effects of major compounds derived from coffee on BP [17–26]. A_1_R—adenosine A_1_ receptor; A_2A_R—adenosine A_2A_ receptor; A_2B_R—adenosine A_2B_ receptor; GFR—glomerular filtration rate; PDE—phosphodiesterase; ACE—angiotensin-converting enzyme; TPR—total peripheral resistance; SOD—superoxide dismutase; CAT—catalase; BP—blood pressure; NO—nitric oxide; NAD(P)H—nicotinamide adenine dinucleotide phosphate oxidase

The most common chemical compounds in coffee are caffeine, chlorogenic acid, trigonelline, kahweol, and cafestol (Figure 1) [3]. Less abundant compounds found in coffee include mannose, polysaccharide chains of galactose, melanoidins, flavonoids, catechins, anthocyanins, ferulic acid, caffeic acid, p-coumaric acid, and tocopherols [9, 11]. Since it is likely that not all of the chemical compounds present in coffee have been identified, and that the mechanisms of action of most of the identified compounds are not yet fully understood, the biological properties of coffee are currently attributed to the effects of the best described compounds, such as caffeine, chlorogenic acid, trigonelline, cafestol, and kahweol, as well as ferulic acid. Figure 2 summarizes the potential biochemical mechanisms of the influence of coffee on BP.

The biologically active compounds of coffee have many mechanisms of action. Caffeine can both increase and decrease BP by antagonizing the adenosine receptors A_1_R, A_2A_R, and A_2B_R, thus altering total peripheral resistance, diuresis, and heart rate. Regular consumption of 2–3 cups of coffee per day leads to the development of tolerance to caffeine. This tolerance explains the lack of a pressor effect of caffeine in people who habitually consume coffee [19]. Other compounds found in coffee, such as chlorogenic acid, trigonelline, melanoidins, and ferulic acid, have antihypertensive effects mediated by reducing angiotensin converting enzyme activity, protecting the vessels against oxidative stress, and increasing the bioavailability of nitric oxide [17–24].

Habitual coffee consumption may also affect BP by altering the composition of the gut microbiota. A study by González et al. assessed the impact of habitual coffee consumption (up to 45 ml/day and 45–500 ml/day) on the composition of the gut microbiota in a population of 147 healthy normotensive persons. Coffee consumption has been shown to be associated with an increase in the level of *Bacteroides* [25]. Two groups of coffee-derived (poly)phenols, methoxyphenols and alkylphenols, as well as caffeine, were positively associated with gut microbiota *Bacteroides* levels [25]. Since the level of *Bacteroides* in gut microbiota is negatively associated with systolic and diastolic BP [26], habitual coffee consumption may lower BP via increasing the level of *Bacteroides* in gut microbiota.

In summary, coffee contains many biologically active compounds, and the content of these compounds varies depending on the type of coffee, the way it is prepared, and many other factors, which have been described above. Compounds contained in coffee, such as chlorogenic acid, ferulic acid, melanoidins, and trigonelline, have antihypertensive effects.

## Coffee Consumption and the Risk of All-Cause and CVD Mortality

A recent study by Torres-Callado et al. assessed the effects of coffee consumption on all-cause, cardiovascular, and cancer mortality. The study included 1567 people who were followed for 18 years. Consumption of > 1 cup of coffee/day was associated with a reduced risk of all-cause mortality (HR = 0.56; 95% CI: 0.41–0.77) and cancer ([HR = 0.41; 95% CI: 0.20–0.86) but had no effect of CVD mortality (HR = 0.71; 95% CI: 0.41–1.20) [27]. In contrast, a meta-analysis by Di Maso et al. of 26 prospective studies showed that consumption of 3–4 cups of coffee/day significantly reduced risk of developing or dying from CVD (RR = 0.90; 95% CI: 0.84–0.96) [8]. In a study by Tverdal et al. of 508,747 persons followed for 20 years, coffee consumption (filtered and unfiltered) was significantly associated with 21% and 16% reductions in risk of all-cause mortality in men and women, respectively. Subgroup analysis showed that coffee consumption was significantly associated with a 28% reduction in the risk of death from CVD in women (women: HR = 0.72; 95% CI: 0.61–0.85 but not in men: HR = 0.93; 95% CI: 0.83–1.04). In men, only the consumption of filtered coffee was associated with a significant 12% reduction in the risk of death due to CVD (HR = 0.88; 95% CI: 0.81–0.96) [28]. A meta-analysis of 31 studies by Grosso et al. showed that consumption of up to 4 cups of coffee a day was associated with a 14% reduction in the risk of all-cause mortality (RR = 0.86; 95% CI: 0.82–0.89) and a 15% reduction in the risk of CVD mortality (RR = 0.85; 95% CI: 0.77–0.93). In addition, non-smokers derived greater benefit from consuming coffee than those who smoked [29].

The effect of coffee consumption on the risk of mortality in patients with pre-existing CVD has also been examined. A study by Teramoto et al. of 46,213 patients, including those with and without an antecedent heart attack or stroke, who were followed for 18.5 years showed a significant 14% reduction in the risk of death in those without prior myocardial infarction or stroke who consumed 1–6 cups of coffee/day (HR = 0.86; 95% CI: 0.82–0.91). There was no significant effect of coffee consumption on the risk of death in patients with a stroke history (HR = 1.31; 95% CI: 0.94–1.82), but there was a significant 31% reduction in risk of death in patients with a history of myocardial infarction who consumed 1–6 cups of coffee/day (HR = 0.69; 95% CI: 0.53–0.91) [30].

Polymorphisms in the gene encoding the enzyme CYP1A2 involved in caffeine metabolism may influence the biological effects of coffee consumption. Increased risk of myocardial infarction and arterial hypertension has been found in individuals who carry a functional variant of cytochrome P450 1A2 (CYP1A2), which makes them less effective in metabolizing caffeine [31–33]. A study by Zhou and Hyppönen that included data from 347,077 individuals in the UK Biobank showed that consuming 1–6 cups of coffee a day was not significantly associated with risk of CVD. Moreover, an analysis of CYP1A2 gene polymorphisms (CYP1A2 AA—fast caffeine metabolism versus CYP1A2 CA + CC—slow caffeine metabolism) showed that polymorphisms in the gene did not affect the observed effects of coffee consumption on CVD risk (p ≥ 0.53) [34].

In summary, coffee consumption can reduce the risk of all-cause and CVD mortality, including in patients after a myocardial infarction. Factors such as smoking or the way coffee is prepared have a significant impact on the observed effect of coffee consumption on human health. Importantly, the biochemical mechanisms of the beneficial effects of coffee consumption in reducing all-cause mortality are not well understood. A recent systematic review of 17 randomized clinical trials by Daneschvar et al. found a lack of convincing evidence that an anti-inflammatory effect of coffee is a major contributing factor to the lower all-cause mortality reported in observational studies of the effects of coffee consumption on CVD risk [35].

## Coffee Consumption and BP and Risk of Arterial Hypertension—Results of Clinical Studies and Meta-analyses

The effects of coffee consumption on BP and risk of hypertension have been examined in many studies and meta-analyses, as summarized in Table 2. Results of these studies and meta-analyzes indicate that the habitual (regular) consumption of 2–3 cups of coffee a day does not alter the risk of arterial hypertension in most people, especially in women and non-smokers. Conversely, non-habitual (irregular; occasional) coffee consumption is associated with an increase in BP and may increase the risk of arterial hypertension.

**Table 2:**
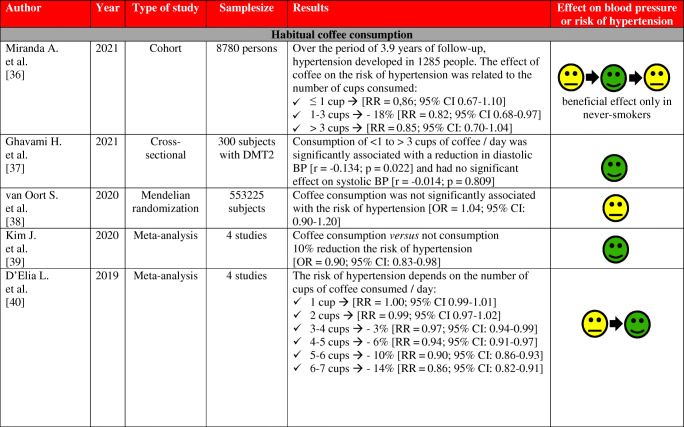

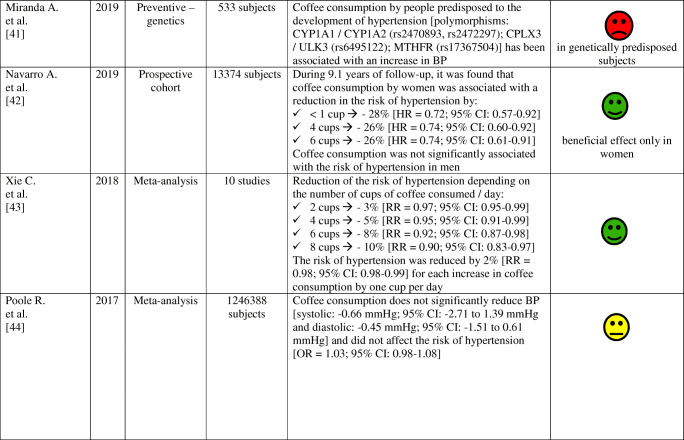

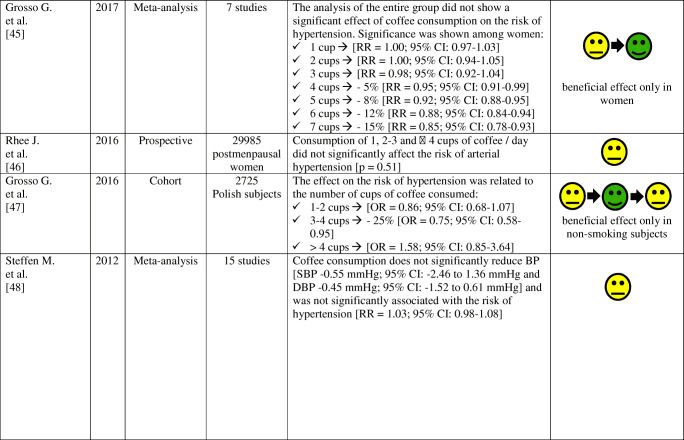

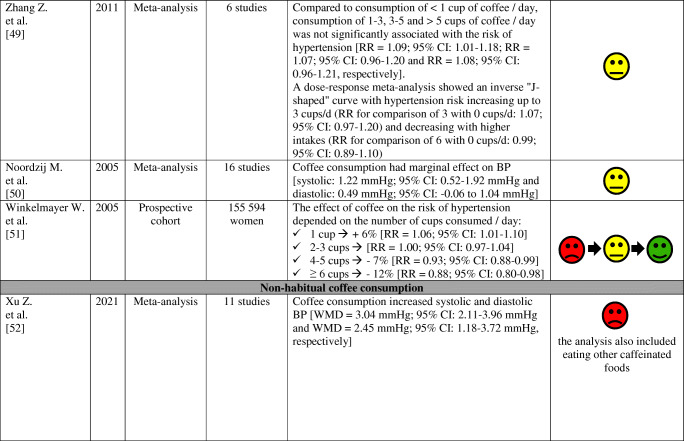

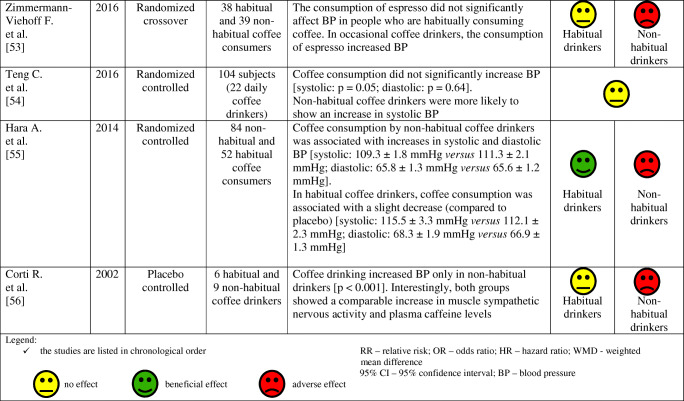
Effect of habitual and non-habitual coffee consumption on blood pressure and the risk of arterial hypertension—summary of studies results and meta-analysis

## Safety of Coffee Consumption by Patients with Arterial Hypertension

The meta-analysis of Mesas et al. analyzed the effects of a single ingestion of 200–300 mg of caffeine (5 studies) and habitual coffee consumption (6 studies) on BP and CVD risk in patients with arterial hypertension. Ingestion of 200–300 mg of caffeine (1.5–2 cups of coffee) increased systolic BP by 8.14 mmHg (95% CI: 5.68–10.61 mmHg) and diastolic BP by 5.75 mmHg (95% CI: 4.09–7.41 mmHg). The duration of the BP effect was at least 3 h. Studies of the longer-term effect (2 weeks) of coffee consumption showed no increase in BP. Habitual coffee consumption has not been shown to increase CVD risk in patients with arterial hypertension [57]. A study by Palatini et al. that analyzed the association of coffee consumption and CYP1A2 polymorphism with risk of impaired fasting glucose in hypertensive patients (n=1180) also provided accurate data on coffee consumption and BP. There were no significant differences in 24-h BP between non-coffee drinkers and those consuming 1–3 and > 3 cups of coffee/day (systolic BP: 130.9±10.4 mmHg versus 131.0±10.8 mmHg versus 131.8±12.0 mmHg (p = 0.72); diastolic BP: 81.5±8.1 mmHg versus 81.5±8.2 mmHg versus 81.0±8.0 mmHg (p = 0.79)) [58].

The HARVEST study enrolled 1,204 participants with arterial hypertension and followed them for 12.6 years. It showed that consumption of 1–3 cups of coffee per day was associated with a non-significant increase in the risk of CVD events (HR = 2.8; 95% CI: 1.0–7.9), while consumption of ≥ 4 cups a day significantly increased the risk (HR = 4.5; 95% CI: 1.4–14.2) [59]. A study by Lopez-Garcia et al. examined the effect of habitual coffee consumption on the risk of uncontrolled 24-h BP (BP ≥ 130/80 mmHg) in elderly patients with arterial hypertension (n = 715). Persons who consumed between 1 and > 3 cups of coffee a day had a higher risk of uncontrolled BP (OR = 1.95; 95% CI: 1.15–3.30 and OR = 2.55; 95% CI: 1.28–5.09). Consumption of 2 cups of coffee/day was not significantly associated with lack of BP control (OR = 1.41; 95% CI: 0.75–2.68). Among women, no significant effect of coffee consumption (1, 2, or > 3 cups/day) on the risk of uncontrolled BP was found. Importantly, after taking into account smoking, the consumption of 1, 2, and > 3 cups of coffee/day in never smokers was not significantly associated with risk of uncontrolling BP. Further, coffee consumption was not significantly associated with the risk of a non-dipper BP profile in either women or men [60].

In summary, the results of these studies indicate that the consumption of 1–3 cups of coffee/day in most patients with arterial hypertension does not increase the risk of uncontrolled BP, including the occurrence of the non-dipper BP profile, or the risk of a CVD event.

## Factors Limiting the Interpretation of Findings of Studies and Meta-analyses

An important limitation of studies of the impact of coffee consumption on human health is the lack of information about what kind of coffee was consumed by their participants (type, blend, country of origin, type of grains, preparation method, additives milk and/or sugar, as well as the different definition of a cup). In the systematic review by Daneschvar et al., it was found that consumption of boiled coffee increased the serum concentration of total cholesterol, low-density lipoprotein, and apolipoprotein B. This effect was not observed in people who consumed filtered coffee [35]. Another significant limitation is the lack of detail about the spectrum of CVD risk factors in study participants. Further, lack of information about the influence of the participants’ diet, as well as polymorphisms of many genes encoding enzymes involved in the metabolism of biochemical components of coffee, as well as their biological actions (e.g., activation of adenosine receptors) is another limitation of published studies. These polymorphisms may differ between the races of study participants, possibly resulting in differential sensitivity to coffee and thus the occurrence of various biological effects after its consumption [61–63]. Recently, an important role of epigenetics has been cited to account for the different effects of coffee observed in various studies since coffee consumption is associated with different levels of DNA methylation in many CpG sites, which can lead to a change in the expression of various genes. Varying levels of CpG methylation may thus explain the different biological effects of coffee consumption observed in various studies [64, 65]. The variable concentrations of biologically active compounds other than caffeine in coffee may also explain the discrepancies observed in some studies and limit the possibility of their interpretation. Moreover, most of the meta-analyses were based on the results of observational studies, which allows only the formulation of hypotheses and not the assessment of cause-and-effect relationships. In addition, there is growing evidence that coffee and smoking can have an interactive effect on blood pressure, as it can have a significant effect on the results of studies. Researchers from the HARVEST Study described this phenomenon as early as 1995 [66]. Importantly, in some studies, no differences in biological effects were observed between natural coffee and decaffeinated coffee, indicating a significant role for constituents other than caffeine.

## Review of Clinical Recommendations and Summary

Table 3 summarizes the opinions of various hypertension/cardiology societies on the impact of coffee consumption on the risk of arterial hypertension.

**Table 3:** Summary of societies’ positions on the influence of coffee consumption on arterial hypertension, CVD, and overall health. *BP* blood pressure, *CVD* cardiovascular disease

Scientific society	Specific comment on effects on BP or CVD	References
International Society of Hypertension (2020)	➢ Moderate consumption of coffee is a healthy drink	[67]
Polish Society of Hypertension (2019)	➢ Available research evidence, mostly of observational nature, does not indicate a higher risk of hypertension development or higher blood pressure values in people who regularly drink coffee	[68–70]
European Society of Hypertension and European Society of Cardiology (2018)	➢ Caffeine has been shown to have an acute pressor effect ➢ Coffee consumption is associated with cardiovascular benefits	[71]
American College of Cardiology, American Heart Association, and American Society of Hypertension (2017)	➢ Coffee use in patients with hypertension is associated with acute increases in BP ➢ Long-term use of coffee is not associated with increased blood pressure or cardiovascular disease	[72]

Coffee is a widely consumed drink all over the world. Coffee contains many biologically active compounds which result in multidirectional effects on the regulation of BP. Regular moderate (1–3 cups of coffee/day) coffee consumption may reduce BP and the risk of developing hypertension, as well as the risk of death from any cause. Habitual and moderate (1–3 cups of coffee/day) coffee consumption likely does not increase the risk of uncontrolled BP and does not disturb the circadian BP profile in hypertensive patients.
